# Plasma Aβ42/40 predicts progression from Aβ-amyloid negative to positive PET scans

**DOI:** 10.1016/j.tjpad.2025.100455

**Published:** 2026-01-01

**Authors:** Azadeh Feizpour, Vincent Doré, Pierrick Bourgeat, James D. Doecke, Rodrigo Canovas, Simon M. Laws, Tenielle Porter, Kun Huang, Christopher Fowler, Ralph N. Martins, Paul Maruff, Hamid R. Sohrabi, Michael W. Weiner, John C. Morris, Tammie L.S. Benzinger, Suzanne E. Schindler, Randall J. Bateman, Yan Li, Ovod Vitaliy, Larry Ward, Jurgen Mejan-Fripp, Colin L. Masters, Victor L. Villemagne, Christopher C. Rowe, ADOPIC Consortium (AIBL, ADNI, OASIS)

**Affiliations:** a Florey Institute of Neuroscience and Mental Health, The University of Melbourne, 30 Royal Parade, Parkville, VIC, 3052, Australia; b Department of Molecular Imaging & Therapy, Austin Health, 145 Studley Road, Heidelberg, VIC, 3084, Australia; c The Australian e-Health Research Centre, CSIRO, 351 Royal Parade, Parkville, VIC, 3052, Australia; d The Australian e-Health Research Centre, CSIRO, 296 Herston Rd, Herston, Qld, 4029, Australia; e Centre for Precision Health, Edith Cowan University, 270 Joondalup Drive, Joondalup, WA, 6027, Australia; f Collaborative Genomics and Translation Group, School of Medical and Health Sciences, Edith Cowan University, 270 Joondalup Drive, Joondalup, WA, 6027, Australia; g Curtin Medical School, Curtin University, Kent Street, Bentley, WA, 6102, Australia; h Australian Alzheimer’s Research Foundation, Nedlands, WA, 6009, Australia; i Cogstate Ltd, 161 Collins St, Melbourne, VIC, 3000, Australia; j Centre for Healthy Ageing, Health Futures Institute, Murdoch University, WA, 6150, Australia; k School of Psychology, Murdoch University, WA, 6150, Australia; l Department of Radiology, University of California at San Francisco, San Francisco, 505 Parnassus Avenue, San Francisco, CA, 94143, USA; m Department of Neurology, Washington University School of Medicine, 660 South Euclid Avenue, St. Louis, MO, 63110, USA; n Charles F. and Joanne Knight Alzheimer Disease Research Center (Knight ADRC), Washington University School of Medicine, 660 South Euclid Avenue, St. Louis, MO, 63110, USA; o Department of Radiology, Washington University in St. Louis, 510 South Kingshighway Boulevard, St. Louis, MO, 63110, USA; p Tracy Family SILQ Center, Washington University School of Medicine, 660 South Euclid Avenue, St. Louis, MO, 63110, USA; q Department of Psychiatry, University of Pittsburgh, 3811 O’Hara Street, Pittsburgh, PA, 15213, USA

**Keywords:** Plasma Aβ42/40, Aβ-amyloid PET, Alzheimer’s disease, Progression risk, Primary prevention trial

## Abstract

**Background:**

The agreement between plasma Aβ42/40 and Aβ positron emission tomography (PET) is approximately 75 %, with ∼85 % of discrepancies due to positive plasma but negative PET results. It is unclear whether this reflects Aβ changes in plasma before PET-detectable.

**Objectives:**

To assess the influence of Aβ42/40 positivity on risk of progression to Aβ PET positivity, and feasibility of using plasma Aβ42/40 tests to enrich a primary prevention trial.

**Design:**

A prospective longitudinal cohort study.

**Setting:**

Participants of Australian Imaging, Biomarkers and Lifestyle study (AIBL), Alzheimer’s Disease Neuroimaging Initiative (ADNI), and Open Access Series of Imaging Studies 3 (OASIS3).

**Participants:**

507 cognitively unimpaired adults at baseline, with a baseline Aβ PET < 20 Centiloid (CL) and available longitudinal Aβ PET data.

**Measurements:**

Baseline Aβ PET and plasma Aβ42/40 measurement by mass-spectrometry, followed by 1–6 additional Aβ PET scans every 1.5–3 years. Those < 5 CL were classified as PET- and 5–20 CL as PET_Low_. Plasma -/+ was defined using the Aβ42/40 Youden’s Index threshold (0.119), corresponding to Aβ PET status.

**Results:**

At baseline, 283 were Plasma-/PET-, 97 Plasma+/PET-, 76 Plasma-/PET_Low_, and 51 Plasma+/PET_Low_. Among Plasma+/PET- individuals, 19 % progressed to PET+ (>20 CL), indicating a higher risk of progression, compared to Plasma-/PET- (HR: 3.90 [90 % CI: 2.00–7.61], *p* < 0.001). This elevated risk remained significant after matching the groups’ baseline CL (3.43 [1.43–8.26], *p* = 0.010), or adjustment for age, sex, *APOE* ε4 and baseline CL (2.48 [1.22 - 5.07], *p* = 0.013). Plasma+/PET- individuals accumulated Aβ ∼8 times faster (1.14 CL/year) than Plasma-/PET- (0.15 CL/year, *p* < 0.001). Plasma+/PET- progressors became PET+ two years earlier than Plasma-/PET- progressors. Among the Plasma+/PET_Low_ individuals, 67 % progressed to PET+. Their progression was faster and earlier than in the Plasma-/PET_Low_ group (HR: 20.82 [11.28 - 38.42], *p* < 0.001 vs. 6.67 [3.51 - 12.65], *p* < 0.001; reference: Plasma-/PET-), largely driven by higher baseline CL in the Plasma+ group. In a primary prevention paradigm targeting high-risk PET_Low_ individuals, pre-screening with Aβ42/40 blood test reduced the number of PET scans by 49 %, compared to a PET-only strategy.

**Conclusions:**

Cognitively unimpaired individuals with abnormal Aβ42/40 are at increased risk for future Aβ PET positivity. In the 5–20 CL subgroup, baseline CL is the main driver of this risk. Combining blood-based pre-screening with PET imaging may help efficiently enrich primary prevention trials.

## 1 Introduction

The amyloid-β (Aβ) peptide, formed from the proteolytic cleavage of amyloid precursor protein (APP), shows lower biofluid levels in Alzheimer’s disease (AD). Extensive research has investigated cerebrospinal fluid (CSF) Aβ levels. Lower baseline CSF Aβ42 levels are associated with future CSF Aβ positivity [1]. A prior study found that individuals with abnormal CSF Aβ42 but normal Aβ PET show increased cortical Aβ accumulation–similar to those with both abnormal CSF and PET results, and higher than those with normal results in both modalities [2]. These findings suggest that CSF changes in Aβ are more sensitive to early stages of Aβ deposition than Aβ PET [3], and changes in CSF Aβ precede change in Aβ PET [4].

Compared to measuring CSF Aβ, measuring plasma Aβ levels has been challenging due to its 50–100 times lower concentration compared to CSF or brain tissue, leading to years of conflicting results [5,6]. However, immunoprecipitation-mass spectrometry (IPMS) and improved immunoassays now enable precise detection of low plasma Aβ quantities [[7], [8], [9]]. The IPMS Aβ assay revealed robust associations (up to *r* = 0.78) with PIB Aβ PET tracer standardized uptake value ratios (SUVR) [8], and areas under curves (AUC) of 0.94 to 0.97 to distinguish Aβ PET+ from Aβ PET- [8]. IPMS-based Aβ42/40 assays have since outperformed most immunoassays in head-to-head comparisons [7,10].

While emerging high-performance plasma biomarkers, particularly tau phosphorylated at threonine 217 (pTau217), demonstrate superior classification of Aβ PET status, Aβ42/40 exhibits a comparatively lower positive predictive value (PPV) [11]. This discrepancy stems from the elevated rate of false positives associated with Aβ42/40 as judged against Aβ PET. Similar to the trajectory of CSF Aβ42/40, a previous longitudinal study has estimated that, the trajectory of plasma Aβ42/40 precedes that of brain Aβ levels by a median of 6 years [12]. This compels a critical examination: Are the Aβ42/40 false positives merely measurement artifacts, or could they represent a more nuanced phenomenon–potentially capturing subtle biofluid Aβ dynamic changes before Aβ accumulation becomes detectable by PET imaging? Addressing such questions is crucial, as the modest cross-sectional accuracy of plasma Aβ42/40 may stem from its earlier emergence compared to PET-detectable Aβ aggregates.

Some current anti-Aβ therapeutic trials target asymptomatic or mildly symptomatic Aβ PET+ individuals [[13], [14], [15], [16]]. However, there may be greater benefit in prevention–intercepting molecular and cellular changes before the emergence of Aβ aggregates. Identifying cognitively unimpaired but at-risk participants remains challenging for primary prevention paradigms due to low Aβ PET positivity rates [17]. However, a subset of individuals is on the cusp of converting to Aβ positivity, underscoring the need to distinguish those closer to pathological onset for timely intervention. If similar to results reported for CSF [[1], [2], [3], [4]], changes in plasma Aβ42/40 are a harbinger of progression to Aβ PET positivity, this accessible and affordable biomarker could enhance prognostic decision-making and refine eligibility criteria for prevention trials.

A previous study [9] found that 30 % of Aβ PET-negative individuals with a positive IPMS-measured plasma Aβ42/40 (<0.1218) progressed to Aβ PET positivity. Our study aims to replicate these findings in a larger, multi-centre cohort, delving deeper to determine whether the progression can truly be predicted by plasma Aβ42/40 positivity.

Our first aim was to examine the risk of progression to Aβ PET positivity in cognitively unimpaired individuals with abnormal plasma Aβ42/40. We took two measures to both adjust for and eliminate the effect of baseline CL in risk prediction using Cox proportional hazards model adjusted for covariates and matching groups’ baseline CL values, respectively. Our second aim was to determine if screening participants with the IPMS Aβ42/40 plasma assay can reduce the number of PET screening scans and enrich for those on the verge of converting to Aβ PET positivity.

The aims of this study were tested using a unique dataset comprised of three large-scale longitudinal cohorts: the Australian Imaging, Biomarkers and Lifestyle study (AIBL) [18], the Alzheimer’s Disease Neuroimaging Initiative (ADNI) [19,20], and the Open Access Series of Imaging Studies 3 (OASIS3) [21], as a part of the Alzheimer's Dementia Onset and Progression in International Cohorts (ADOPIC) study.

## 2 Methods

### 2.1 Participants

The study cohort represents a convenience sample of 507 cognitively unimpaired (CU) participants from the AIBL (*n* = 220) [18], the ADNI, (*n* = 91) [19,20], and the OASIS3 (*n* = 196) [21]. A brief description of each cohort’s recruitment process, eligibility criteria and ethical approval is provided in Supplementary Table 1. Briefly, included participants 1) were CU at baseline, as determined by neuropsychological assessments within the normative range for their age group or based on a combination of age, sex, and educational background; 2) had baseline plasma Aβ42/40 results available from IPMS assays; 3) had Aβ PET < 20 CL at baseline; and 4) had two or more Aβ PET scans conducted more than seven months apart. Study participants self-reported sex.

The main analyses focused exclusively on participants with Aβ PET < 20 CL at baseline. However, for illustrative purposes, the last section focused on primary prevention trial workflow includes an additional cohort (*n* = 159) with Aβ PET > 20 CL at baseline to demonstrate the effect of different CL categories in a hypothetical primary prevention trial design.

### 2.2 Ethics

Each study was approved by the Institutional Review Boards of their respective participating institutions. Written informed consents were obtained from all participants, prior to undergoing study procedures (see Supplementary Table 1 for cohort-specific details).

### 2.3 Plasma Aβ42/40 collection and analysis

For the AIBL cohort, blood samples were obtained following an overnight fast and collected in 7.5 mL EDTA tubes containing the anticoagulant along with an added prostaglandin E1 (PGE1) supplement (Sapphire Biosciences, 33.3 ng/mL). Blood samples from participants in the ADNI study were collected after an overnight fast and drawn into 10 mL K2-EDTA tubes that did not contain PGE1, and these samples were subsequently centrifuged at 1300 g for a duration of 10 min. For the OASIS cohort, non-fasted participants provided blood samples which were collected in 15 mL EDTA tubes. These tubes were gently inverted to mix the contents and then centrifuged at a low speed to sediment any cellular debris. Plasma samples from OASIS were assayed for the targeted Aβ isoforms (Aβ42 and Aβ40) by C_2_N Diagnostics, as per procedures previously described [22], and samples from AIBL and ADNI were assayed at the Bateman Lab, Washington University, as per protocols previously described [23]. To correct for batch (cohort) effects, batch harmonization was performed using the batchtma package in R [24], ensuring that all groups achieved a mean equivalent to the grand mean of the three cohorts. This method adjusts for batch effects in biomarker data; however, it does not account for differences in mass spectrometry methodologies, or preanalytical sample handling (e.g., fasting status, tube type, time to centrifugation, or inclusion of PGE1).

To define plasma status, Youden’s index was used to determine the threshold at which plasma Aβ42/40 maximized the discrimination of Aβ PET status. This analysis was performed on a larger sample including both cognitively unimpaired and impaired participants across AIBL, ADNI and OASIS (*n* = 1089). A plasma Aβ42/40 ratio < 0.119 was considered positive. This threshold yielded sensitivity of 0.81 [0.70 - 0.84], specificity of 0.74 [0.73 - 0.86], PPV of 0.69 [0.67 - 0.78], NPV of 0.85 [0.80 - 0.87] and accuracy of 0.77 [0.76 - 0.81]. The plasma Aβ42/40 Youden’s index threshold was the same (0.119) if only cognitively unimpaired participants were included.

### 2.4 Aβ PET imaging and analysis

Aβ PET imaging was performed at each respective cohort site. AIBL Aβ PET scans were acquired using ^11^C-PiB (PiB), ^18^F-Florbetapir (FBP), ^18^F-NAV4694 (NAV), or ^18^F-Flutemetamol (FLUTE), and ADNI and OASIS3 Aβ PET scans were acquired using PiB or FBP.

For PET quantification, we used the SPM pipeline, as described by the Centiloid consortium [25]. Briefly, each PET image was rigidly aligned to their matching T1-weighted MRI. The T1W MRI was then affinely and non-rigidly aligned to the Montreal Neurological Institute (MNI) template. Each PET image was non-rigidly deformed using the T1W MRI deformation field. PET quantification was ultimately performed utilizing Centiloid masks in the normalised space. Images were normalised using the whole cerebellum. Due to use of different PET scanners across the three studies and at different timepoints of each study, PET images were smoothed to a uniform 8 mm full width half-maximum point spread function using the methodology of Joshi et al. (2009) [26]. This is the standardised processing pipeline used to transform SUVR into Centiloid for each available tracer (Standard Centiloid).

When combining Aβ PET measurements from different centres and timepoints, standard Centiloid conversion does not fully eliminate differences due to varying protocols, techniques and tracers [27]. We have previously demonstrated that quantification using the Non-negative Matrix Factorisation (NMF) method enhances inter-tracer agreement, effect size, and longitudinal consistency [28,29]. Therefore, due to tracer changes in the majority of participants across different time points, NMF-based quantification was applied to all spatially normalised and scaled images. This method decomposes each PET image into specific and non-specific binding components using a two-component NMF decomposition and provides better consistency across tracers [29]. Because other groups and labs use the Standard Centiloid, we have also included some results calculated based on the Standard Centiloid method in the Supplementary Material.

### 2.5 Aβ PET thresholds

For progression to Aβ PET positivity (PET+), a threshold of 20 Centiloid (CL) was used. Such positivity threshold is consistent with that used in the secondary AHEAD 3–45 prevention trial, for inclusion of those with intermediate Aβ (≈20 to 40 CL for A3) [14]. We have also previously shown that this threshold had the highest accuracy in detecting moderate-frequent neuritic plaque density [30].

A baseline CL cut point of 5 was used to define PET negative (PET-) participants. This approach was adopted to minimize the influence of baseline CL on the estimated risk of progression to Aβ PET positivity, enabling a more rigorous assessment of the impact of plasma Aβ42/40 status on the risk of progression. This threshold was determined based on a receiver operating characteristic (ROC) analysis, which included all participants with a baseline Aβ PET < 20 CL, and at least three consecutive Aβ PET scans (*n* = 487). The analysis demonstrated that a baseline CL threshold of ∼5 provided a negative predictive value (NPV) ∼90 % for identifying participants who remained stable (i.e., did not progress above 20 CL, across the median follow up of 7.46 years (IQR: 5.78–9.21)).

### 2.6 Plasma/PET status

#### 2.6.1 Plasma-/PET- and Plasma+/PET-

For the primary analysis, we assessed the impact of plasma Aβ42/40 positivity on risk of progression to PET positivity, after minimising the influence of baseline PET by only including those with < 5 CL. Hence, two groups were defined: plasma Aβ42/40 negative & Aβ PET negative (Plasma-/PET-, reference group, *n* = 283) and plasma Aβ42/40 positive & Aβ PET negative (Plasma+/PET-, *n* = 97).

#### 2.6.2 Plasma-/PET-, plasma-/pet_low_, plasma+/ PET_Low_

For the secondary analysis, we evaluated the impact of plasma Aβ42/40 positivity combined with presence of low Aβ burden (5–20 CL), on risk of progression to PET positivity. Therefore, three groups were considered: Plasma-/PET- (reference group, *n* = 283), plasma Aβ42/40 negative, Aβ PET in low range (5–20 CL) (Plasma-/PET_Low_, *n* = 76) and plasma Aβ42/40 positive, Aβ PET in low range (Plasma+/PET_Low_, *n* = 51).

### 2.7 Statistical analyses

All analyses were performed using Python (version 3.9.16), except batch effect-adjusting of Aβ42/40 data received from three different centres for which we used batchma library in R with “Simple means” approach [24]. Between-group comparisons for quantitative features were performed using a Mann-Whitney U rank test when the data were non-normally distributed and independent T-test when normally distributed. Normality was assessed using the Shapiro-Wilk test. Categorical variables (sex, Apolipoprotein E (*APOE)* ε4, and progression to Aβ PET+) were compared using the chi-square (χ²) test. Values are reported as median (interquartile range, IQR) due to non-normal distribution. To simplify *APOE* genotyping, we recoded the variable to a binary format, where individuals with no ε4 allele were assigned a value of 0, and those carrying one or two ε4 alleles were assigned a value of 1. For sensitivity, specificity, positive predictive values (PPV), NPV, and accuracy, the 90 % confidence intervals (90 % CI, shown in square brackets) were calculated by using 1000 bootstrap replicates (without replacement to reduce bias). The 90 % CI were then computed using the percentile method.

To examine progression to Aβ PET+, we used the Kaplan-Meier method. Differences between survival curves were tested by pairwise log-rank tests, with false discovery rate correction (for comparisons involving three curves). We derived hazard ratios (HR) from Cox proportional-hazards (CPH) models, with progression to Aβ PET+ as the outcome, and with and without adjustments for age, sex, *APOE* ε4 carriership and baseline CL, using Plasma-/PET- as the main reference group. Due to the limited number of “at risk” participants beyond 11 years, longitudinal data for all groups were right-censored at 11 years before being input into the Kaplan-Meier and CPH model. The Cox proportional-hazards over time assumption was confirmed using the Schoenfeld residuals method for the primary analysis, but not for the secondary analysis (the effect of Plasma/PET status varied by time). As per the recommendation by Stensrud and Hernán (2020) [31], we still reported the HR for the secondary analysis, but recommend interpreting it as a weighted average of the varying true hazard ratios over the entire follow-up period. We also address the limitation of using Cox regression models when the HR is not constant during the follow-up period, by reporting bootstrapped 90 % CI, as per Stensrud and Hernán (2020) [31]. Due to the smaller sample size of the comparison groups (relative to the reference group: Plasma-/PET-), we added a condition to the bootstrapping process to ensure that only randomly drawn samples with > 30 observations were included.

Kaplan-Meier method and CPH models were performed for two CL ranges: first, for those with Aβ PET < 5 CL (PET-), and second, for those with Aβ PET between 5 and 20 CL (PET_Low_). The first analysis aimed to examine the risk of progression to Aβ PET positivity in cognitively unimpaired individuals with abnormal plasma Aβ42/40, while minimizing the impact of baseline CL. The second analysis aimed to assess whether the influence of elevated plasma Aβ42/40 levels acted synergistically with, or was masked by, the presence of sub-threshold Aβ accumulation. For the rationale behind choosing 5 CL to separate this analysis, please refer to the ‘Aβ PET thresholds’ section.

Additionally, we took several measures to both adjust for (using CPH models adjusted for baseline CL and other covariates) and eliminate (matching groups by baseline CL values) the effect of baseline CL in risk prediction in both sets of analyses. Nearest-neighbour matching was implemented in Python using the KDTree algorithm, which identified, for each participant in one group, the nearest baseline Centiloid match from the other group.

To calculate annual rate of change in CL, a linear model was fitted to each participant’s CL values, over two to seven timepoints independently and the regression slopes were reported as accumulation rate. Group CL trajectories were modelled using a locally weighted scatterplot smoothing (LOWESS) method.

## 3 Results

### 3.1 Participants

Among the 507 included participants, 283 were Plasma-/PET-, 97 were Plasma+/PET-, 76 were Plasma-/PET_Low_, and 51 were Plasma+/PET_Low_. Across all participants, the mean (± SD) follow up was 6.9 ± 3.0 years. Compared to Plasma-/PET- participants, Plasma+/PET- participants had a lower proportion of females (*p* = 0.002), higher percentage of *APOE* ε4 carriers (*p* = 0.003) and slightly higher baseline CL (*p* = 0.021). Sex or *APOE* genotype did not affect Plasma-/PET_Low_ and Plasma+/PET_Low._ Baseline CL was slightly higher in the Plasma+/PET_Low_ group (*p* = 0.03). The level of plasma Aβ42/40 was lower and proportion of individuals progressing to Aβ PET+ was higher in both Plasma+/PET- (compared to Plasma-/PET-) and Plasma+/PET_Low_ (compared to Plasma-/PET_Low_) groups (both *p* < 0.001). The follow-up duration did not differ significantly between the groups (Table 1). For a breakdown by cohorts (AIBL, ADNI and OASIS), see Supplementary Tables 2, 3 and 4, respectively.

**Table 1:** Participant characteristics.

	Plasma-/PET-	Plasma+/PET-	p	Plasma-/PET_Low_	Plasma+/PET_Low_	p
*N* = 507	283	97	__	76	51	__
Age, years	68.76 (63.88–74.31)	70.62 (66.54–73.95)	0.129	70.48 (67.24–74.56)	72.47 (67.48–77.05)	0.267
Sex, %Female (N)	60 % (171)	42 %[41]	0.002	59 %[45]	49 %[25]	0.258
*APOE*, %ε4 (N)	15 %[43]	29 %[28]	0.003	18 %[14]	33 %[17]	0.055
Baseline Centiloid	−3.2 (−6.74–0.26)	−2.34 (−5.93–2.08)	0.021	9.28 (7.3–11.59)	10.86 (7.82–15.47)	0.03
Plasma Aβ42/40	0.13 (0.12–0.13)	0.11 (0.11–0.12)	<0.001	0.13 (0.12–0.13)	0.11 (0.11–0.12)	<0.001
Progression to Aβ PET+ (N)	6 %[17]	19 %[18]	<0.001	34 %[26]	67 %[34]	<0.001
Time from baseline to first detection of PET+, years	8.99 (7.36–9.79)	6.32 (5.32–8.41)	0.083	3.59 (2.42–6.62)	3.66 (2.06–5.47)	0.399
Follow-up, years	6.55 (4.19–9.22)	6.41 (4.97–8.97)	0.962	7.94 (4.71–9.39)	7.88 (5.94–9.27)	0.896

Values presented as counts, Median (IQR) or percentages (number). Plasma-/PET-: plasma Aβ42/40 ≥ 0.119 & PET < 5 CL; Plasma+/PET-: plasma Aβ42/40 < 0.119 & PET < 5 CL; Plasma-/PET_Low_: plasma Aβ42/40 ≥ 0.119 & PET between 5 and 20 CL; Plasma+/ PET_Low_: plasma Aβ42/40 < 0.119 & PET between 5 and 20 CL. Third column demonstrates p values of Mann-Whitney U rank test, the Kruskal-Wallis test or independent T-test for Plasma-/PET- vs. Plasma+/PET-. Sixth column lists p values of Mann-Whitney U rank test or the Kruskal-Wallis test for Plasma-/PET_Low_ vs. Plasma+/PET_Low_. Progression to Aβ PET+ and Follow-up years are based on all longitudinal observations up to 14 years. Time from baseline to first detection of PET+ indicates when the event was first observed, not necessarily when it occurred (calculated for the subset of each cohort who progressed to PET+). *APOE*: Apolipoprotein E.

For those with abnormal Aβ42/40 and Aβ PET < 5 CL (Plasma+/PET-), 19 % progressed to Aβ PET positive (>20 CL) while among those with normal Aβ42/40 and Aβ PET < 5 CL (Plasma-/PET-), 6 % progressed to Aβ PET positive. The median (IQR) time from baseline to detection of first Aβ PET positivity was 6.32 (5.32–8.41) and 8.99 (7.36–9.79) years, respectively.

In those with abnormal Aβ42/40 and Aβ PET between 5 and 20 CL (Plasma+/PET_Low_), 67 % progressed to Aβ PET positive during the follow up period while among those with normal Aβ42/40 and PET between 5 and 20 CL (Plasma-/PET_Low_), 34 % progressed. The median (IQR) time from baseline to detection of first PET positivity was 3.66 (2.06–5.47) and 3.59 (2.42–6.62) years, respectively.

### 3.2 Outcomes in participants with baseline PET < 5 CL (PET-)

Individuals with baseline PET < 5 CL were examined to determine their risk of progression to Aβ PET positivity while minimizing the confounding effects of baseline CL. The rationale for using 5 CL as the threshold is detailed in the 'Aβ PET thresholds' section of the Methods.

#### 3.2.1 Progression to Aβ PET positivity in plasma+ vs. Plasma- groups with PET < 5 CL

**Fig. 1:**
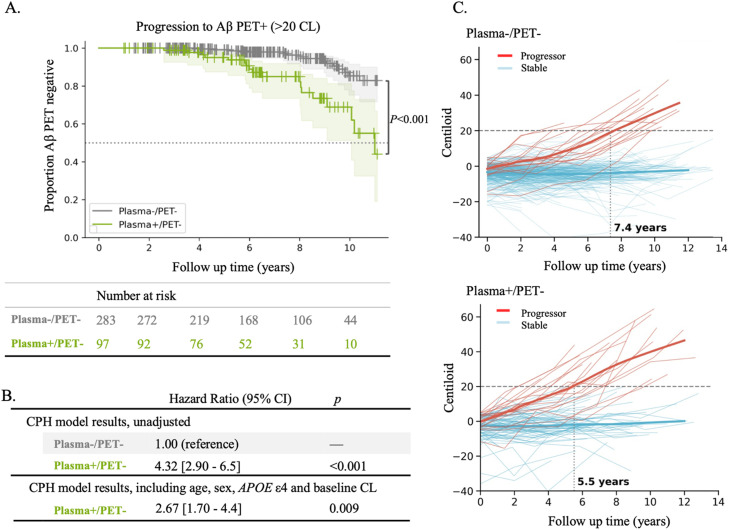
Progression to Aβ PET+ in Plasma+/PET- (*n* = 97) vs. Plasma-/PET- (*n* = 283) groups. A. Survival curves for progression to Aβ PET+ in Plasma+/PET- vs. the reference group, Plasma-/PET-. Table below the figure indicates the total number of participants at risk at each time point. Plasma-/+ based on Aβ42/40 threshold of 0.119. PET- was defined as < 5CL. *P* value indicates result of a log-rank test between Kaplan-Meier survival curves, for the entire 11 years. B. Hazard Ratios and bootstrapped 90 % confidence intervals from Cox proportional hazard models, presented as both unadjusted and adjusted for age, sex, *APOE* ε4, and baseline Centiloid. C. Longitudinal plot of Centiloid value changes over time for Plasma-/PET- and Plasma+/PET-, whereby progressors (those who progressed to > 20 CL) are plotted in red and stable participants (those who remained below 20 CL) are plotted in blue. A locally weighted scatterplot smoothing (LOWESS) curve was fitted to the longitudinal data using the nonparametric LOWESS method. Years at which progressors became PET+ are annotated. PET- is defined as PET less than 5 CL.

The Kaplan-Meier survival curves for Plasma-/PET- vs. Plasma+/PET- participants are shown in Fig. 1A, with the Plasma+ group showing a faster progression to PET+. The Kaplan-Meier survival data, including the number of censored observations and events (progression to >20 CL) at key time points, are detailed in Supplementary Tables 5 and 6. The log-rank test indicated a significant difference in survival curves between Plasma-/PET- and Plasma+/PET- participants over the entire 11-year period (*p* < 0.001). When the analysis was restricted to five years, the difference remained significant (*p* = 0.024).

Cox proportional-hazards model showed an increased risk for future progression to Aβ PET+ in the Plasma+/PET- group compared to the Plasma-/PET- (reference) group (HR = 4.32, [90 % CI = 2.90 - 6.5.7], P < 0.001; Fig. 1B). After adjusting for age, sex, *APOE* ε4 and baseline CL, the hazard ratio decreased but remained significant (HR = 2.67 [1.70 - 4.4], *p* = 0.009; Fig. 1B). Similar results were found when using Standard Centiloid (as opposed to NMF) (Supplementary Figure 1A-B).

Next, we case-matched the baseline CL of the Plasma-/PET- to the Plasma+/PET- groups to ensure that neither group was at a more advanced disease stage at the start of the study (Supplementary Figure 2). After matching the baseline CL values, the percentage of Plasma-/PET- who progressed increased to 9 % (from 6 % before matching). There was still a significant difference in survival curves between Plasma-/PET- and Plasma+/PET- participants over the entire 11-year period (*p* = 0.003) and the unadjusted hazard ratio was 3.43 [2.16 - 6.1] (*p* = 0.006). The difference between survival curves was borderline significant when the analysis was restricted to 5 years (*p* = 0.049). These findings suggest that the observed difference in progression to PET+ between the Plasma+ and Plasma- group is independent of baseline CL, when baseline CL is below 5.

#### 3.2.2 Aβ accumulation rate and longitudinal changes in centiloid

The mean Aβ accumulation rate was 1.14 CL/year for Plasma+/PET-, almost 8 times that for Plasma-/PET- (0.15 CL/year, *p* < 0.001). Comparing the progressors in these two groups, the mean Aβ accumulation rates was 4.97 CL/year for Plasma+/PET- progressors vs. 3.61 CL/year for Plasma-/PET- progressors (*p* = 0.003).

Plotting longitudinal Centiloid changes over time for each group (Fig. 1C) indicated that the progressors (those who progressed to > 20 CL at any time across the 14-year follow up) among the Plasma+/PET- participants became abnormal on Aβ PET at 5.5 years, almost 2 years earlier than progressors in the Plasma-/PET- group (at 7.5 years). Notably, Plasma+/PET- progressors had baseline CL levels comparable to those of Plasma-/PET- progressors (CL: Median 0.96 (IQR: −3.01 - 3.41) vs. −0.99 ((−4.42 - 1.3), respectively, *p* = 0.255). For baseline demographics of progressors vs. stables in each Plasma/PET group, see Supplementary Table 7.

Using Standard Centiloid revealed a 3.2-year lag between Plasma+/PET- progressors and Plasma-/PET- progressors in becoming abnormal on PET (Supplementary Figure 1C).

### 3.3 Outcomes in participants with baseline PET between 5 and 20 CL (PET_Low_)

This section focuses on individuals with baseline PET of 5–20 CL to evaluate whether the effect of positive plasma Aβ42/40 was synergistic with, or overshadowed by, the effect of sub-threshold Aβ burden.

#### 3.3.1 Progression to Aβ PET positivity in plasma+ vs. Plasma- groups with PET between 5 and 20 CL

**Fig. 2:**
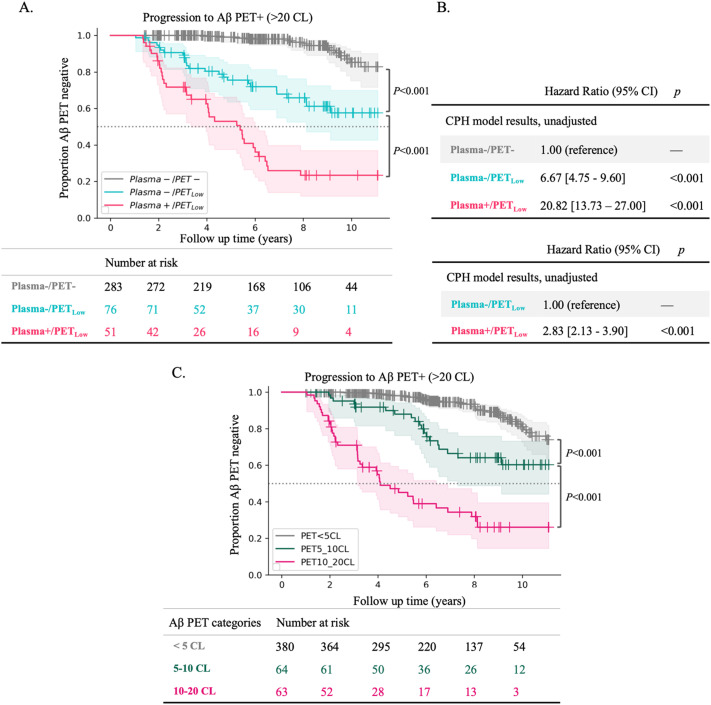
Progression to Aβ PET+ in Plasma+/PET_Low_ (*n* = 51) vs. Plasma-/PET_Low_ (*n* = 76) groups, along with groups defined based on Centiloid categories. A. Survival curves for progression to Aβ PET+ in Plasma-/PET_Low_ and Plasma+/PET_Low_ vs. Plasma-/PET-. Table below the figure indicates the total number of participants at risk at each time point. Plasma-/+ based on Aβ42/40 threshold of 0.119. PET- defined as < 5CL. PET_Low_ defined as CL between 5 and 20. P value indicates result of a pairwise log-rank test between Kaplan-Meier survival curves, for the entire 11 years, with false discover rate correction. B. Hazard Ratios and bootstrapped 90 % confidence intervals from unadjusted Cox proportional hazard models, for Plasma-/PET_Low_ and Plasma+/PET_Low_ when using Plasma-/PET- as the reference; as well as for Plasma+/PET_Low_ when using Plasma-/PET_Low_ as the reference. C. Survival curves for progression to Aβ PET+ in groups defined by baseline CL categories (regardless of plasma A42/40 values): < 5 CL (*n* = 380), 5–10 CL (*n* = 64), and 10–20 CL (*n* = 63).

Fig. 2A shows the Kaplan-Meier survival curves for Plasma-/PET-, Plasma-/PET_Low_, and Plasma+/PET_Low_ participants. The Plasma+/PET_Low_ group exhibited a faster and earlier decline in survival probability compared to the Plasma-/PET_Low_ group, which in turn declined faster than the Plasma-/PET- group. The Kaplan-Meier survival data, including the number of censored observations, and events (progression to >20 CL) at key time points, are detailed in Supplementary Table 8 and 9. The pairwise log-rank tests indicated a significant difference in survival curves between Plasma-/PET- participants and Plasma-/PET_Low_ (*p* < 0.001), as well as between Plasma-/PET_Low_ and Plasma+/PET_Low_ participants (*p* < 0.001), over the entire 11-year period. When the analysis was restricted to 5 years, the difference remained significant (*p* < 0.001 for Plasma-/PET- vs. Plasma-/PET_Low_ and *p* = 0.008 for Plasma-/PET_Low_ vs. Plasma+/PET_Low_).

Cox proportional-hazards model showed a heightened risk for future progression to Aβ PET+ in the Plasma-/PET_Low_ group compared to the Plasma-/PET- reference group (HR =  6.67 [4.75 - 9.60], P < 0.001), for Plasma+/PET_Low_ compared to the Plasma-/PET- reference group (HR =  20.82 [13.73 - 27.00], P < 0.001) and for Plasma+/PET_Low_ compared to Plasma-/PET_Low_ reference group (HR =  2.83 [2.13 - 3.90], P < 0.001) (Fig. 2B).

Importantly, after matching the baseline CL values of Plasma-/PET_Low_ and Plasma+/PET_Low_, pairwise log-rank test showed that the difference between their survival curves over the entire 11-year period decreased to borderline significant (*p* = 0.048). Additionally, the unadjusted hazard ratio reduced to 1.67 [1.25 - 2.3] which was not significant (*p* = 0.058) (Supplementary Figure 3B). This suggests that plasma positivity, with PET in the 5–20 CL range, could indicate a considerably steep trajectory of progression to Aβ PET positivity but, this effect is driven by higher baseline CL values in the Plasma+/PET_Low_ group.

To further test this hypothesis, regardless of plasma Aβ42/40 values, we categorized baseline CL values into three groups: < 5 CL, 5–10 CL, and 10–20 CL. We then plotted Kaplan-Meier survival curves for each group to assess progression to PET+ (>20 CL) (Fig. 2C). As illustrated, the decline in survival probability for the Plasma+/PET_Low_ group closely resembled that of the 10–20 CL category, highlighting the substantial impact of baseline CL levels on the risk of progression, beyond the influence of plasma Aβ42/40.

### 3.4 Prevention trial enrichment

For prevention trials aimed at slowing or halting Aβ accumulation, it is crucial to identify cognitively unimpaired, Aβ PET negative individuals who are 1) at high risk of progressing to Aβ PET positivity and 2) progress to PET+ within a short timeframe. In the previous sections, we established that 67 % of Plasma+/PET_Low_ individuals progressed to Aβ PET positivity with the median time to detection of positivity being 3.66 years (IQR: 2.06–5.47) (See Table 1). This is a suitable cohort for prevention trials, due to high conversion rate within a relatively short period of time. Below, we examine practicality of enriching a trial with such high-risk participants, compare three strategies and discuss possible challenges.

#### 3.4.1 Strategy A. two-step process: pre-screening with plasma Aβ42/40, followed by aβ PET for plasma+ cases

**Fig. 3:**
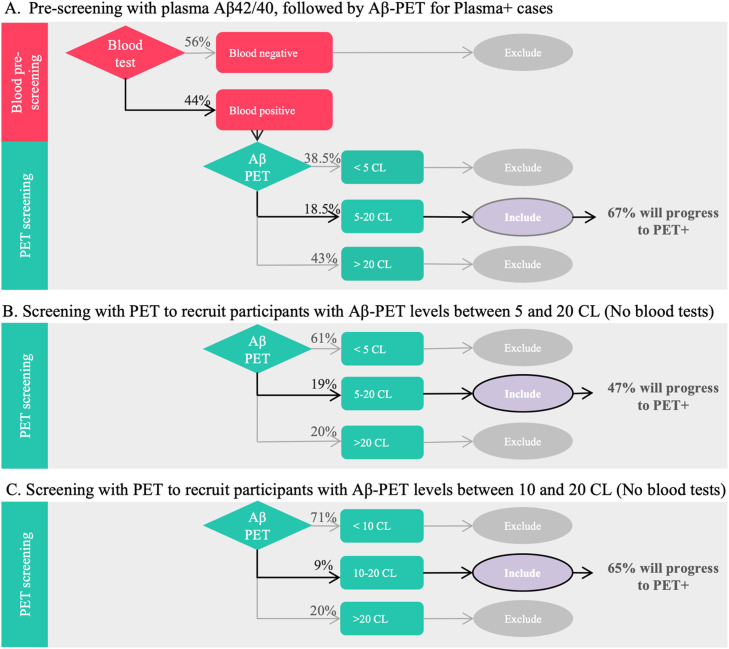
Identification of high-risk cohorts for prevention trials. A. Two-step process (step1: blood test, step 2: Aβ PET scan) for refining the participant pool to identify a biomarker-based cohort at the highest risk of progression. Among cognitively unimpaired individuals pre-screened with a blood test, 44 % had abnormal plasma Aβ42/40 (Plasma+), of which 18.5 % had Aβ PET levels between 5 and 20 CL (PET_Low_). This strategy identifies the Plasma+/PET_Low_ group who are at the highest risk for progression to Aβ PET positive (with 67 % progressing). B. An alternative strategy involves using PET alone. However, defining the 5–20 CL range based solely on PET, without blood test pre-screening, results in a cohort where only 47 % will progress to Aβ PET positivity. C. A third strategy involves using PET alone, but this time including only those with PET between 10 and 20 CL to enrich for progressors. This results in a cohort where 65 % will progress to Aβ PET positivity, comparable to the prevalence achieved with the two-step process. For a comparison of number of blood tests and/or PET scans, see Table 2.

Fig. 3A illustrates a two-step process. Step 1: pre-screening with the IPMS Aβ42/40 blood test, followed by Step 2: Aβ PET screening of Plasma+ individuals to identify those who are 5–20 CL. As shown in Table 2, to achieve a target number of high-risk participants (denoted by N), we need to pre-screen almost 12 times that number (12.3 × *N*) using Aβ42/40 blood test. Of these pre-screened individuals, 44 % would be Plasma+. Among the Plasma+ individuals (5.4 × *N*), 18.5 % would have PET levels between 5 and 20 CL (Plasma+/PET_Low_). Given our previous findings that 67 % of Plasma+/PET_Low_ individuals would progress to Aβ PET positivity, with the median time to detection of positivity being 3.66 years, this cohort may be suitable for prevention trials.

**Table 2:** Estimated number of blood tests and/or PET scans to include high-risk cognitively unimpaired in a prevention trial.

No. of Blood tests	No. of PET scans	Target sample
**Strategy A.** Two-Step Process: Pre-screening with plasma Aβ42/40, followed by Aβ PET for Plasma+ cases:		
12.3 × *N* (44 % of which will be Blood positive)	5.4 × *N* (18.5 % of which will have Aβ PET between 5 and 20 CL)	N (67 % of which are expected to progress to Aβ PET+)

**Strategy B.** Screening with Aβ PET to recruit 400 CU with Aβ PET between 5 and 20 CL:		
	5.3 × *N* (19 % of which will have Aβ PET between 5 and 20 CL)	N (47 % of which are expected to progress to Aβ PET+)

**Strategy C.** Screening with Aβ PET to recruit 400 CU with Aβ PET between 10 and 20 CL to enrich for progressors:		
	11.1 × *N* (9 % of which will have Aβ PET between 10 and 20 CL)	N (65 % of which are expected to progress to Aβ PET+)

Comparison of three recruitment strategies for high-risk participants. The number of PET scans will be 49 % lower if blood pre-screening is done prior to PET screening (Strategy A) compared to Strategy C. Strategy A and C are compared as the target number and prevalence of converters within the target cohort are similar. N represents the total number of high-risk participants for a prevention trial aimed at preventing the formation of Aβ plaques.

#### 3.4.2 Strategy B: screening with PET to recruit participants with Aβ PET levels between 5 and 20 CL (No blood tests)

Fig. 3B illustrates the impact of eliminating the blood pre-screening stage. As shown in Table 2, to achieve N high-risk participants, we need to screen 5.3 × *N* with Aβ PET, 19 % of whom would have Aβ PET levels between 5 and 20 CL (PET_Low_). However, our data presented in Table 2 showed that only 47 % of PET_Low_ individuals would progress to PET positivity (with the median time to detection of positivity being 3.64 years (IQR: 2.1–5.75)), making this group less comparable to Strategy A.

#### 3.4.3 Strategy C: same as strategy B, but this time including those with aβ PET levels between 10 and 20 CL (No blood tests)

To enhance the prevalence of progressors, we defined a 10–20 CL category (Fig. 3C). As shown in Table 2, to achieve N high-risk participants, we need to screen 11.1 × *N* with Aβ PET, 9 % of whom would have Aβ PET levels between 10 and 20 CL (PET_10–20CL_). Our data showed that 65 % of PET_10–20CL_ would progress to PET positivity with the median time to detection of positivity being 3.15 years (IQR: 2.01–4.51). This is a comparable conversion rate to that for Strategy A.

#### 3.4.4 Comparison of the two successful strategies (A and C)

Strategy A (Two-step process) required 5.4 × *N* Aβ PET scans to achieve a high-risk cohort with a 67 % conversion rate, while Strategy C required 11.1 × *N* Aβ PET scans for a similar outcome (65 % conversion rate). Including the blood pre-screening step in the Two-step process reduced the required PET scans by 49 %. This makes the Two-step process the most effective strategy for identifying cognitively unimpaired individuals at the highest risk of progressing to Aβ positivity. However, it is important to note that regardless of the strategy, the target cohort constitutes only 8–9 % of the pre-screened/screened cohort, highlighting the challenge for prevention trials.

## 4 Discussion

To evaluate whether plasma Aβ42/40 positivity despite negative Aβ PET predicts future Aβ PET positivity, we retrospectively analysed 507 cognitively unimpaired participants from a large-scale multicentre study with high-precision plasma Aβ42/40 and up to 14 years of follow-up PET data. We found that: 1) cognitively unimpaired individuals who were plasma Aβ42/40 positive had a higher risk of progressing to Aβ PET positivity and among those who were plasma positive and had baseline PET between 5–20 CL (Plasma+/PET_Low_), 67 % progressed to Aβ PET positivity (>20 CL) during follow-up; 2) enriching for prevention trial with Plasma+/PET_Low_ individuals could reduce the number of required PET scans by 49 %.

Due to the confounding effect of baseline CL in predicting progression to Aβ PET positivity, we stratified analyses into two sections: one for participants with baseline PET < 5 CL and another for those with PET between 5 and 20 CL. In the first section, we assessed the effect of plasma Aβ42/40 more independently of baseline CL. In the latter section, we evaluated whether the effect of positive plasma Aβ42/40 was synergistic with, or overshadowed by, the effect of low Aβ burden.

First, we observed that among participants with Aβ PET <5 CL, 25 % had abnormal plasma Aβ42/40 at baseline; 19 % of these progressed to PET positivity (>20 CL) over 14 years, though this may be underestimated due to attrition by year 6. We also showed that abnormal level of plasma Aβ42/40 was a significant risk factor for future PET positivity, when baseline CL influence was minimised. This supports its role in capturing early, subtle biofluid Aβ alterations before PET can detect Aβ accumulation, albeit not in all participants. Interestingly, it was previously reported that in participants with Aβ PET ≤ 20 CL, the strongest correlation observed between low levels of Aβ PET and leading plasma biomarkers (pTau181, pTau217, Aβ42/40, GFAP, and NfL) was with plasma Aβ42/40 [11]. These findings underscore the value of plasma Aβ42/40 as a suitable marker for identifying individuals at risk before PET positivity is reached.

Second, among individuals with subthreshold Aβ PET (5–20 CL), those with abnormal plasma Aβ42/40 exhibited a faster and earlier decline in probability of remaining Aβ PET negative, compared to those with normal Aβ42/40 levels. This initially suggested a synergistic effect of subthreshold-level CL and plasma Aβ42/40. However, it was later determined that the primary driver was the higher CL level in those with abnormal levels of Aβ42/40. Therefore, within the Aβ PET range of 5–20 CL, plasma Aβ42/40 did not add predictive value beyond baseline Aβ PET.

IPMS-based plasma Aβ42/40 assays predict current Aβ positivity (PET or CSF) with AUCs of 0.80–0.88 [9,23,32,33], and a recent head-to-head comparison shows they outperform most immunoassays [7], which explains the current study's focus on IPMS-based Aβ42/40. Such superior performance led to the initial use of IPMS (C2N) plasma Aβ42/40 in the AHEAD 3–45 trial [14] to pre-screen for *current* amyloid positivity. Due to its high NPV, it was used to exclude participants unlikely to be eligible based on PET imaging, reducing the number of PET scans and thus screening costs [34]. Later, plasma %p-tau217 was added to the biomarker algorithms, to improve classification accuracy [35]. These developments highlight the limitations of Aβ42/40 in detecting *current* Aβ PET positivity and suggest that while %p-tau217 may be more effective for pre-screening in trials targeting *current* pathology, plasma Aβ42/40 could serve as a valuable predictor of *future* amyloid positivity due to its earlier trajectory.

Consistent with our findings, one study of 74 Aβ PET-negative (≤1.42 for PiB and ≤1.22 for FBP) individuals, with ∼4 years of follow up, showed that those with a positive IPMS plasma Aβ42/40 had a 15-fold increase in risk of progression to Aβ PET-positive compared to individuals with a negative plasma Aβ42/Aβ40 [9]. However, these findings were based on a relatively small sample size of 51 Plasma-/PET- and 23 Plasma+/PET- individuals, among which only eight progressed to PET positive.

We also demonstrated that Plasma+/PET- progressors (who were < 5 CL at baseline) became abnormal on Aβ PET (> 20 CL) on average after 5.5 years, acknowledging a margin of error as the exact onset of plasma positivity is unknown. This aligns with a previous longitudinal study estimating that the trajectory of plasma Aβ preceded that of brain Aβ by a median of 6 years [12].

The clinical significance of our findings lies in addressing the challenge of interpreting discordant cases, where plasma Aβ42/40 levels are abnormal but Aβ PET results are normal. Our study demonstrated that a positive plasma result, despite a negative PET, is a risk factor for future progression to PET positivity. However, if accurate PET quantification techniques are available to the clinicians, baseline Centiloid value could both indicate risk of progression and inform the timing of the follow up scan. Therefore, plasma Aβ42/40 is unlikely to be suitable as a standalone biomarker for determining therapy eligibility in routine clinical practice. It is noteworthy that aside from future PET positivity, expectedly cognitive decline does not appear to be a short-term concern for these patients. A previous report suggested that the longitudinal cognitive trajectory of Plasma+ individuals with a negative PET closely resembled that of Plasma- individuals with a negative PET, over a maximum 6.6-year period [36]. Moreover, we have previously demonstrated that in cognitively unimpaired individuals, cognitive decline due to AD was not seen when baseline CL values were below 25 and was minimal when CL values were below 50, over 6 years of follow up [37].

Next, we considered implication of our findings in the context of prevention trials. Recent therapeutic trials have started using anti-Aβ antibodies at an earlier stage in the disease course, such as A4 (solanezumab) [16], AHEAD 3–45 (lecanemab) [14], and TRAILBLAZER-ALZ-3 (donanemab) for preclinical AD [13], targeting asymptomatic or mildly symptomatic participants with evidence of Aβ pathology. The emphasis on earlier intervention stems from irreversible damage at the later stages, likely due to the presence of cortical tau usually observed at Aβ levels 50 CL [38], as well as the higher prevalence of comorbidities that contribute to cognitive impairment with advancing age. Most individuals diagnosed later in life have Alzheimer's disease in combination with cerebrovascular or other neurodegenerative comorbidities such as Lewy body disease [39]. However, some argue that intervention should occur even earlier-preventing the development of the hallmark lesions of Alzheimer's disease [40], and that it is the only way to prove or refute the Aβ-amyloid hypothesis [30]. Individuals with dominantly inherited Alzheimer's disease (DIAD), caused by mutations in PSEN1, PSEN2, or APP, are the ideal population to evaluate the efficacy of anti-Aβ therapies at an early stage [41]. However, DIAD is a rare disorder, comprising less than 1 % of AD cases [42] and the generalizability of such findings for the more common “sporadic” late-onset Alzheimer's disease is challenging. This underscores the necessity for tools and strategies that allow effective identification of cognitively unimpaired individuals who are at high risk for developing sporadic AD pathology. Our findings in cognitively unimpaired individuals with subthreshold Aβ PET Centiloid levels (5–20 CL) revealed that plasma Aβ42/40 was not more informative than baseline Centiloid for identifying high-risk participants. With PET quantification techniques readily available in clinical trials, baseline Centiloid measurements can effectively identify at-risk cohorts. However, incorporating plasma Aβ42/40 as a pre-screening measure could reduce the number of required PET scans by 49 %. Despite this, the enduring challenge of screening for prevention trials remains: the low prevalence of conversion to PET positivity among cognitively unimpaired individuals.

Strengths of this study include the consolidation of three of the largest longitudinal cohort studies, with long-term follow-up, enabling a robust evaluation of longitudinal changes in CL for groups stratified by plasma Aβ42/40 status. A limitation of this study is the low diversity in the AIBL, ADNI, and OASIS cohorts. Consequently, it is unclear whether the predictive ability of plasma Aβ42/40 varies by racial and ethnic diversity. Another limitation is the unique inclusion of IPMS Aβ42/40 assay results in this study. Whether these findings also apply to other plasma Aβ42/40 immunoassays warrants further investigation. Adding *APOE* ε4 to predict the risk of progression was not investigated in this study due to sample size restrictions. Additionally, while ε4 carriership could enrich the target cohort with fast or early progressors, it would also focus the cohort on individuals most at risk of ARIA in an anti-Aβ trial. Plasma pTau217 was not investigated in this study, as the research question centred around discrepancies between plasma Aβ42/40 and Aβ PET. Additionally, we have previously shown that pTau217 has a similarly strong correlation with both Aβ (Spearman’s r of 0.76) and tau (Spearman’s r of 0.78) PET and high positive predictive values (0.93 for Aβ PET status) [43], potentially showing a closer temporal relationship with these neuroimaging markers than plasma Aβ42/40, and therefore proving more suitable for predicting *current* pathological status rather than *future* PET positivity. A further limitation relates to preanalytical variability in plasma collection and processing protocols across cohorts. For example, the AIBL cohort used prostaglandin E1 (PGE1) to prevent platelet activation, while ADNI and OASIS did not. Fasting status also varied, with AIBL and ADNI participants fasted and OASIS non-fasted. However, recent evidence suggests that fasting affects absolute Aβ concentrations but not the Aβ42/40 ratio [44]. Additionally, sample handling protocols-such as time to centrifugation and freezing-differed across cohorts and were not uniformly documented. As reported previously [45], such factors can influence Aβ42 and Aβ40 concentrations, and using the Aβ42/40 ratio does not fully eliminate these effects. While batch harmonization was applied to reduce inter-cohort variability, residual bias may remain and should be considered when interpreting results.

In conclusion, cognitively unimpaired individuals who test positive for plasma Aβ42/40 are at a higher risk of progressing to Aβ PET positivity in the future. This finding highlights the potential of plasma Aβ42/40 as a valuable biomarker in screening for clinical trials focused on preventing the onset of Aβ positivity.

## Declaration of generative AI and AI-assisted technologies

During the preparation of this work the author(s) used Microsoft Copilot in order to improve readability of pre-written text. After using this tool/service, the author(s) reviewed and edited the content as needed and take(s) full responsibility for the content of the published article.

## Funding

This work was supported by the National Health and Medical Research Council [GA16788], the National Institutes of Health [R01-AG058676–01A1], and the National Institute on Aging grant [R01AG070941]. The sponsors had no role in the design and conduct of the study; in the collection, analysis, and interpretation of data; in the preparation of the manuscript; or in the review or approval of the manuscript.

## CRediT authorship contribution statement

**Azadeh Feizpour:** Conceptualization, Methodology, Software, Formal analysis, Investigation, Writing - Original Draft, Writing - Review & Editing, Visualization. **Vincent Doré:** Conceptualization, Methodology, Validation, Writing - Review & Editing. **Pierrick Bourgeat:** Methodology, Software, Validation, Data Curation, Writing - Review & Editing. **James D. Doecke:** Data Curation, Writing - Review & Editing. **Rodrigo Canovas:** Data Curation, Writing - Review & Editing. **Simon M. Laws:** Resources, Writing - Review & Editing. **Tenielle Porter:** Resources, Writing - Review & Editing. **Kun Huang:** Data Curation, Writing - Review & Editing. **Christopher Fowler:** Resources, Writing - Review & Editing. **Ralph N. Martins:** Resources, Writing - Review & Editing. **Paul Maruff:** Writing - Review & Editing. **Hamid R. Sohrabi:** Writing - Review & Editing. **Michael W. Weiner:** Conceptualization, Methodology, Writing - Review & Editing, Funding acquisition. **John C. Morris:** Funding acquisition, Writing - Review & Editing. **Tammie L. S. Benzinger:** Writing - Review & Editing. Suzanne **E. Schindler:** Writing - Review & Editing. **Randall J. Bateman:** Writing - Review & Editing. **Yan Li:** Resources, Writing - Review & Editing. **Ovod Vitaliy:** Resources, Writing - Review & Editing. **Larry Ward:** Funding acquisition, Writing - Review & Editing. **Jurgen Fripp:** Conceptualization, Writing - Review & Editing. **Colin L. Masters:** Funding acquisition, Writing - Review & Editing. **Victor L. Villemagne:** Conceptualization, Methodology, Writing - Review & Editing. **Christopher C. Rowe:** Project administration, Supervision, Writing - Review & Editing.

## Declaration of competing interest

CCR has received research grants from NHMRC, Enigma Australia, Biogen, Eisai and Abbvie. He is on the scientific advisory board for Enigma and has consulted for Prothena, Eisai, Eisai Australia, Roche, and Eli Lilly Australia, Nova Nordisk Australia. VLV has received several grants from NIA/NIH and is and has been a consultant or paid speaker at sponsored conference sessions for Eli Lilly, Life Molecular Imaging, ACE Barcelona, and BRI Japan. SML is a scientific advisor for Cytox Ltd. SES has served on scientific advisory boards on biomarker testing and clinical care pathways for Eisai and Novo Nordisk and has received speaking fees for presentations on biomarker testing from Eisai, Eli Lilly, and Novo Nordisk. CLM reports *Ad Hoc* consultancy speaking engagements and scientific advice with Actinogen, Acumen, Alterity, Biogen, Eisai, Eli-Lilly, Roche. PM is an employee of Cogstate Ltd. The other authors did not report any conflict of interest.

## Appendix Supplementary materials

Image, application 1
