# Supplementary material for: Plasma Aβ42/40 predicts progression from Aβ-amyloid negative to positive PET scans
Source: J Prev Alzheimers Dis. 2026 Jan 1;13(2):100455. doi: 10.1016/j.tjpad.2025.100455 (PMC12869057; doi:10.1016/j.tjpad.2025.100455)
Supplement: Supplementary file 1 [file mmc1.docx]

Supplementary Table 1. Cohort descriptions

| Cohort | Description |
| --- | --- |
| AIBL | The Australian Imaging, Biomarker & Lifestyle Flagship Study of Ageing (AIBL) is a longitudinal, prospective cohort study conducted across two sites in Melbourne and Perth. The study was approved by the institutional human research ethics committees at Austin Health, St Vincent’s Health, Hollywood Private Hospital as well as Edith Cowan University. Recruitment was facilitated by advertising to the general community and referrals from memory disorder specialists. All participants gave written informed consent before participating in assessments. The study was conducted in accordance with the Helsinki Declaration of 1975. Study inclusion criteria encompassed participants who were: 1. aged 60 years or older; 2. free from neurological or psychiatric disorders, substance abuse histories, or unstable medical conditions; and 3. classified as cognitively unimpaired (CU) at baseline, by a multidisciplinary review panel, who comprehensively evaluated available cognitive and clinical data, and were blind to PET imaging and blood assays results. This panel comprised neuropsychologists, old age psychiatrists, psychologists, and at times a neurologist and geriatrician (Fowler, Rainey-Smith et al. 2021). |
| ADNI | Data used in the preparation of this article were obtained from the Alzheimer's Disease Neuroimaging Initiative (ADNI) database (adni.loni.usc.edu). The ADNI represents a collaborative research network of academic institutions and medical centres spanning the United States and Canada. Its primary objective is to establish and standardize neuroimaging methodologies and biomarker procedures. The ADNI study was conducted according to Good Clinical Practice guidelines, the Declaration of Helsinki, US 21CFR Part 50 – Protection of Human Subjects, and Part 56 – Institutional Review Boards, and pursuant to state and federal HIPAA regulations. Written informed consent was obtained from all participants and/or authorized representatives and study partners. Study eligibility criteria included: 1. Age 55-90 years; 2. Education level (6 grades or equivalent); 3. Language fluency (English or Spanish); 4. Not being on psychoactive medications affecting cognitive function; 5. Good general health with adequate visual and auditory acuity, no medical contraindications to MRI, Hachinski Ischemic Score ≤ 4 and Geriatric Depression Scale score < 6; 6. Were classified as cognitively unimpaired (at baseline) (Petersen, Aisen et al. 2010).  The ADNI was launched in 2003 as a public-private partnership, led by Principal Investigator Michael W. Weiner, MD. The original goal of ADNI was to test whether serial magnetic resonance imaging (MRI), positron emission tomography (PET), other biological markers, and clinical and neuropsychological assessment can be combined to measure the progression of mild cognitive impairment (MCI) and early Alzheimer's disease (AD). The current goals include validating biomarkers for clinical trials, improving the generalizability of ADNI data by increasing diversity in the participant cohort, and to provide data concerning the diagnosis and progression of Alzheimer’s disease to the scientific community. For up-to-date information, see adni.loni.usc.edu. |
| OASIS3 | Open Access Series of Imaging Studies 3 (OASIS3) is a publicly available cohort study developed by the Knight ADRC and its affiliated studies, which includes participants at various stages of cognitive decline. Participants were recruited from the community via word of mouth, flyers, and community engagements. The participants were consented into Knight ADRC-related projects following procedures which were approved by the Institutional Review Board of Washington University School of Medicine. Study eligibility criteria included: 1. Age 42.5-95.6 years; 2. CDR (Clinical Dementia Rating) score ≤1; 3. Women who were not pregnant or breastfeeding; 4. Did not have implanted medical devices such as pacemakers and drug pump, history or risk of metal in the eye and history of claustrophobia; 5. Were classified as cognitively unimpaired (at baseline) (LaMontagne, Benzinger et al. 2019). |

Supplementary Table 2. Participant characteristics for AIBL cohort

|  | **Plasma-/PET-** | **Plasma+/PET-** | **p** | **Plasma-/PET_Low_** | **Plasma+/PET_Low_** | **p** |
| --- | --- | --- | --- | --- | --- | --- |
| N=220 | 113 | 36 | __ | 48 | 23 | __ |
| Age, years | 72.69 (68.67-76.55) | 72.44 (69.98-76.71) | 0.488 | 72.09 (68.52-75.03) | 72.95 (69.19-77.05) | 0.256 |
| Sex, %Female (N) | 64% (72) | 47% (17) | 0.079 | 60% (29) | 48% (11) | 0.317 |
| *APOE*, %ε4 (N) | 12% (14) | 25% (9) | 0.068 | 10% (5) | 26% (6) | 0.088 |
| Baseline Centiloid | -3.21 (-6.32-0.36) | -2.34 (-5.36-3.24) | 0.253 | 9.25 (7.46-10.58) | 9.95 (7.35-14.01) | 0.256 |
| Plasma Aβ42/40 | 0.13 (0.12-0.14) | 0.11 (0.11-0.12) | <0.001 | 0.13 (0.12-0.13) | 0.11 (0.11-0.12) | <0.001 |
| Progression to Aβ PET+ (N) | 9% (10) | 22% (8) | 0.032 | 21% (10) | 57% (13) | 0.003 |
| Time from baseline to first detection of PET+, years | 9.21 (8.06-9.86) | 7.02 (5.68-10.1) | 0.177 | 3.21 (3.14-6.95) | 3.15 (1.7-5.39) | 0.369 |
| Follow-up, years | 8.15 (5.67-9.52) | 8.69 (5.69-9.84) | 0.634 | 8.9 (5.8-9.78) | 8.55 (6.05-9.72) | 0.902 |

Values presented as counts, Median (IQR) or percentages (number). Plasma-/PET-: plasma Aβ42/40 ≥ 0.119 & PET < 5 CL; Plasma+/PET-: plasma Aβ42/40 < 0.119 & PET < 5 CL; Plasma-/PET_Low_: plasma Aβ42/40 ≥ 0.119 & PET between 5 and 20 CL; Plasma+/ PET_Low_: plasma Aβ42/40 < 0.119 & PET between 5 and 20 CL. Third column demonstrates p values of Mann-Whitney U rank test, the Kruskal-Wallis test or independent T-test for Plasma-/PET- vs. Plasma+/PET-. Sixth column lists p values of Mann-Whitney U rank test or the Kruskal-Wallis test for Plasma-/PET_Low_ vs. Plasma+/PET_Low_. Progression to Aβ PET+ and Follow-up years are based on all longitudinal observations up to 14 years. Time from baseline to first detection of PET+ indicates when the event was first observed, not necessarily when it occurred (calculated for the subset of each cohort who progressed to PET+). *APOE*: Apolipoprotein E.

Supplementary Table 3. Participant characteristics for ADNI cohort

|  | **Plasma-/PET-** | **Plasma+/PET-** | **p** | **Plasma-/PET_Low_** | **Plasma+/PET_Low_** | **p** |
| --- | --- | --- | --- | --- | --- | --- |
| N=91 | 48 | 18 | __ | 12 | 13 | __ |
| Age, years | 73.91 (69.59-78.02) | 72.15 (68.5-75.45) | 0.467 | 69.44 (67.28-74.31) | 73.68 (70.81-79.4) | 0.314 |
| Sex, %Female (N) | 42.0% (20) | 28.0% (5) | 0.3 | 58.0% (7) | 38.0% (5) | 0.32 |
| *APOE*, %ε4 (N) | 21.0% (10) | 22.0% (4) | 0.902 | 25.0% (3) | 23.0% (3) | 0.91 |
| Baseline Centiloid | -4.82 (-13.3--0.69) | -3.01 (-7.31-2.84) | 0.14 | 11.81 (8.78-14.3) | 12.97 (10.27-17.47) | 0.43 |
| Plasma Aβ42/40 | 0.13 (0.12-0.14) | 0.11 (0.11-0.12) | <0.001 | 0.13 (0.12-0.14) | 0.11 (0.11-0.12) | <0.001 |
| Progression to Aβ PET+ (N) | 12.0% (6) | 39.0% (7) | 0.016 | 58.0% (7) | 92.0% (12) | 0.047 |
| Time from baseline to first detection of PET+, years | 6.37 (4.74-8.62) | 8.02 (6.32-8.3) | 0.473 | 4.51 (2.05-5.3) | 3.18 (2.15-4.15) | 0.773 |
| Follow-up, years | 7.27 (4.0-9.93) | 7.64 (5.65-9.71) | 0.458 | 5.81 (3.63-7.98) | 7.88 (7.01-9.08) | 0.053 |

Values presented as counts, Median (IQR) or percentages (number). Plasma-/PET-: plasma Aβ42/40 ≥ 0.119 & PET < 5 CL; Plasma+/PET-: plasma Aβ42/40 < 0.119 & PET < 5 CL; Plasma-/PET_Low_: plasma Aβ42/40 ≥ 0.119 & PET between 5 and 20 CL; Plasma+/ PET_Low_: plasma Aβ42/40 < 0.119 & PET between 5 and 20 CL. Third column demonstrates p values of Mann-Whitney U rank test, the Kruskal-Wallis test or independent T-test for Plasma-/PET- vs. Plasma+/PET-. Sixth column lists p values of Mann-Whitney U rank test or the Kruskal-Wallis test for Plasma-/PET_Low_ vs. Plasma+/PET_Low_. Progression to Aβ PET+ and Follow-up years are based on all longitudinal observations up to 14 years. Time from baseline to first detection of PET+ indicates when the event was first observed, not necessarily when it occurred (calculated for the subset of each cohort who progressed to PET+). *APOE*: Apolipoprotein E.

Supplementary Table 4. Participant characteristics for OASIS cohort

|  | **Plasma-/PET-** | **Plasma+/PET-** | **p** | **Plasma-/PET_Low_** | **Plasma+/PET_Low_** | **p** |
| --- | --- | --- | --- | --- | --- | --- |
| N=196 | 122 | 43 | __ | 16 | 15 | __ |
| Age, years | 62.57 (55.37-67.47) | 67.94 (61.68-71.76) | 0.002 | 65.23 (57.98-69.65) | 67.22 (57.79-70.93) | 0.621 |
| Sex, %Female (N) | 65.0% (79) | 44.0% (19) | 0.018 | 56.0% (9) | 60.0% (9) | 0.833 |
| *APOE*, %ε4 (N) | 16.0% (19) | 35.0% (15) | 0.007 | 38.0% (6) | 53.0% (8) | 0.376 |
| Baseline Centiloid | -3.08 (-5.89--0.43) | -2.19 (-5.95-1.42) | 0.162 | 8.79 (6.42-11.59) | 10.17 (7.23-15.1) | 0.277 |
| Plasma Aβ42/40 | 0.13 (0.12-0.13) | 0.12 (0.11-0.12) | <0.001 | 0.13 (0.12-0.13) | 0.11 (0.11-0.12) | <0.001 |
| Progression to Aβ PET+ (N) | 1.0% (1) | 7.0% (3) | 0.024 | 56.0% (9) | 60.0% (9) | 0.833 |
| Time from baseline to first detection of PET+, years | 11.31 | 4.15 (3.85-4.19) | __ | 3.93 (3.01-6.89) | 5.55 (3.16-6.15) | 0.93 |
| Follow-up, years | 5.96 (3.4-7.93) | 5.96 (3.37-6.49) | 0.524 | 6.85 (3.99-8.34) | 5.92 (3.67-7.53) | 0.54 |

Values presented as counts, Median (IQR) or percentages (number). Plasma-/PET-: plasma Aβ42/40 ≥ 0.119 & PET < 5 CL; Plasma+/PET-: plasma Aβ42/40 < 0.119 & PET < 5 CL; Plasma-/PET_Low_: plasma Aβ42/40 ≥ 0.119 & PET between 5 and 20 CL; Plasma+/ PET_Low_: plasma Aβ42/40 < 0.119 & PET between 5 and 20 CL. Third column demonstrates p values of Mann-Whitney U rank test, the Kruskal-Wallis test or independent T-test for Plasma-/PET- vs. Plasma+/PET-. Sixth column lists p values of Mann-Whitney U rank test or the Kruskal-Wallis test for Plasma-/PET_Low_ vs. Plasma+/PET_Low_. Progression to Aβ PET+ and Follow-up years are based on all longitudinal observations up to 14 years. Time from baseline to first detection of PET+ indicates when the event was first observed, not necessarily when it occurred (calculated for the subset of each cohort who progressed to PET+). *APOE*: Apolipoprotein E.

Supplementary Table 5. Data from Kaplan-Meier curve for Plasma-/PET-

| Time (Years) | Censored | Event |
| --- | --- | --- |
| 0-1 | 0 | 0 |
| 1-2 | 11 | 0 |
| 2-3 | 21 | 0 |
| 3-4 | 31 | 1 |
| 4-5 | 22 | 1 |
| 5-6 | 26 | 2 |
| 6-7 | 42 | 0 |
| 7-8 | 18 | 2 |
| 8-9 | 28 | 3 |
| 9-10 | 26 | 5 |
| 10-11 | 11 | 1 |
| 11+ | 32 | 0 |

The number of censored observations (data missed to follow up) and events (progression to >20 CL) are presented at key time points, for the Plasma-/PET- group.

Supplementary Table 6. Data from Kaplan-Meier curve for Plasma+/PET-

| Time (Years) | Censored | Event |
| --- | --- | --- |
| 0-1 | 1 | 0 |
| 1-2 | 4 | 0 |
| 2-3 | 3 | 1 |
| 3-4 | 11 | 1 |
| 4-5 | 5 | 2 |
| 5-6 | 13 | 4 |
| 6-7 | 14 | 2 |
| 7-8 | 5 | 0 |
| 8-9 | 10 | 4 |
| 9-10 | 6 | 1 |
| 10-11 | 3 | 3 |
| 11+ | 4 | 0 |

The number of censored observations (data missed to follow up) and events (progression to >20 CL) are presented at key time points, for the Plasma+/PET- group.

Supplementary Table 7. Baseline demographics of progressors vs. stables in PET- groups, broken down by plasma status

|  | **Plasma-/PET- stable** | **Plasma-/PET- progressor** | ***p*** | **Plasma+/PET- stable** | **Plasma+/PET- progressor** | ***p*** |
| --- | --- | --- | --- | --- | --- | --- |
| *N=380* | 266 | 17 | __ | 79 | 18 | __ |
| *Age, years* | 68.69 (63.14-74.23) | 71.33 (68.46-76.22) | 0.069 | 70.23 (65.59-73.37) | 71.69 (69.37-75.49) | 0.105 |
| *Sex, %Female (N)* | 60.0 (159) | 71.0 (12) | 0.377 | 39.0 (31) | 56.0 (10) | 0.206 |
| *APOE, %ε4 (N)* | 14.0 (36) | 41.0 (7) | 0.002 | 23.0 (18) | 56.0 (10) | 0.006 |
| *Baseline Centiloid* | -3.39 (-6.85--0.32) | -0.99 (-4.42-1.3) | 0.074 | -2.45 (-6.72-1.42) | 0.96 (-3.01-3.41) | 0.024 |
| *Plasma Aβ42/40* | 0.13 (0.12-0.13) | 0.12 (0.12-0.13) | 0.104 | 0.11 (0.11-0.12) | 0.11 (0.11-0.12) | 0.025 |

Supplementary Table 8. Data from Kaplan-Meier curve for Plasma-/PET_Low_

| Time (Years) | Censored | Event |
| --- | --- | --- |
| 0-1 | 0 | 0 |
| 1-2 | 1 | 4 |
| 2-3 | 3 | 3 |
| 3-4 | 6 | 7 |
| 4-5 | 4 | 3 |
| 5-6 | 6 | 2 |
| 6-7 | 1 | 2 |
| 7-8 | 3 | 1 |
| 8-9 | 8 | 2 |
| 9-10 | 8 | 1 |
| 10-11 | 6 | 0 |
| 11+ | 5 | 0 |

The number of censored observations (data missed to follow up) and events (progression to >20 CL) are presented at key time points, for the Plasma-/PET_Low_ group.

Supplementary Table 9. Data from Kaplan-Meier curve for Plasma+/PET_Low_

| Time (Years) | Censored | Event |
| --- | --- | --- |
| 0-1 | 0 | 0 |
| 1-2 | 2 | 7 |
| 2-3 | 0 | 7 |
| 3-4 | 5 | 4 |
| 4-5 | 0 | 4 |
| 5-6 | 0 | 6 |
| 6-7 | 1 | 5 |
| 7-8 | 0 | 1 |
| 8-9 | 4 | 0 |
| 9-10 | 1 | 0 |
| 10-11 | 1 | 0 |
| 11+ | 3 | 0 |

The number of censored observations (data missed to follow up) and events (progression to >20 CL) are presented at key time points, for the Plasma+/PET_Low_ group.

Supplementary Figure 1. Progression to Aβ PET+ in Plasma+/PET- vs. Plasma-/PET- group, this time using Standard Centiloid

Assessment of progression to Aβ PET+ using Standard Centiloid **A.** Survival curves for progression to Aβ PET+ in Plasma+/PET- vs. the reference group, Plasma-/PET-. Table below the figure indicates the total number of participants at risk at each time point. Plasma-/+ based on Aβ42/40 threshold of 0.119. PET- defined as < 5CL. *P* value indicates result of a log-rank test between Kaplan-Meier survival curves, for the entire 11 years. **B.** Hazard Ratios and bootstrapped 90% confidence intervals from Cox proportional hazard models, presented as both unadjusted and adjusted for age, sex, *APOE* ε4, and baseline Centiloid. **C.** Longitudinal plot of Centiloid value changes over time for Plasma-/PET- and Plasma+/PET-, whereby Progressors (those who progressed to > 20 CL) are plotted in red and Stable participants (those who remained below 20 CL) are plotted in blue. A locally weighted scatterplot smoothing (LOWESS) curve was fitted to the longitudinal data using the nonparametric LOWESS method. Years at which Progressors became PET+ are annotated.

Supplementary Figure 2. Comparison of survival curves and CPH results, before and after matching the baseline Centiloid values

**A.** Survival curves for progression to Aβ PET+ in Plasma+/PET- vs. the reference group, Plasma-/PET-. Table below the figure indicates the total number of participants at risk at each time point. Plasma-/+ based on Aβ42/40 threshold of 0.119. PET- defined as < 5CL. *P* value indicates result of a log-rank test between Kaplan-Meier survival curves, for the entire 11 years. **B.** Repetition of illustrations in panel A. this time including a subset of Plasma-/PET- with similar baseline Centiloid values to Plasma+/PET- (matched samples size of n= 97). Hazard Ratios and bootstrapped 90% confidence intervals from unadjusted Cox proportional hazard models.

Supplementary Figure 3. Comparison of survival curves and CPH results, before and after matching the baseline Centiloid values

**A.** Survival curves for progression to Aβ PET+ in Plasma-/PET_Low_ and Plasma+/PET_Low_ vs. Plasma-/PET-. Table below the figure indicates the total number of participants at risk at each time point. Plasma-/+ based on Aβ42/40 threshold of 0.119. PET- defined as < 5CL. PET_Low_ defined as CL between 5 and 20. *P* value indicates result of a log-rank test between Kaplan-Meier survival curves, for the entire 11 years. Hazard Ratio and bootstrapped 90% confidence interval from unadjusted Cox proportional hazard models, for Plasma+/PET_Low_ when using Plasma-/PET_Low_ as the reference. **B.** Repetition of illustrations in panel A. this time including a subset of Plasma-/PET_Low_ with similar baseline Centiloid values to Plasma+/PET_Low_ (matched samples size of n= 51). Hazard Ratio and bootstrapped 90% confidence interval from unadjusted Cox proportional hazard models.

## **References**

Fowler, C., S. R. Rainey-Smith, S. Bird, J. Bomke, P. Bourgeat, B. M. Brown, S. C. Burnham, A. I. Bush, C. Chadunow and S. Collins (2021). "Fifteen years of the Australian Imaging, Biomarkers and Lifestyle (AIBL) study: progress and observations from 2,359 older adults spanning the spectrum from cognitive normality to Alzheimer’s disease." Journal of Alzheimer's disease reports **5**(1): 443-468.

LaMontagne, P. J., T. L. S. Benzinger, J. C. Morris, S. Keefe, R. Hornbeck, C. Xiong, E. Grant, J. Hassenstab, K. Moulder, A. G. Vlassenko, M. E. Raichle, C. Cruchaga and D. Marcus (2019). "OASIS-3: Longitudinal Neuroimaging, Clinical, and Cognitive Dataset for Normal Aging and Alzheimer Disease." medRxiv: 2019.2012.2013.19014902.

Petersen, R. C., P. S. Aisen, L. A. Beckett, M. C. Donohue, A. C. Gamst, D. J. Harvey, C. R. Jack, Jr., W. J. Jagust, L. M. Shaw, A. W. Toga, J. Q. Trojanowski and M. W. Weiner (2010). "Alzheimer's Disease Neuroimaging Initiative (ADNI): clinical characterization." Neurology **74**(3): 201-209.
